# RNF213 in Panvascular Disease: A Molecular Hub Linking Genetic Susceptibility to Systemic Vasculopathy

**DOI:** 10.3390/biomedicines14081748

**Published:** 2026-08-03

**Authors:** Zhenghaonan Qiu, Guicheng Kuang, Hang Ji, Xinyao Feng, Kunhao Wu, Haogeng Sun, Yi Liu

**Affiliations:** 1 Department of Neurosurgery, West China Hospital, Sichuan University, Chengdu 610041, China; 2 West China School of Medicine, Sichuan University, Chengdu 610041, China

**Keywords:** RNF213, panvascular disease, moyamoya disease, hypoxia-inflammation cycle, endothelial dysfunction, second-hit hypothesis, ubiquitination

## Abstract

Panvascular diseases, characterized by systemic vascular dysfunction across multiple organ systems, represent a complex interplay of genetic susceptibility and environmental triggers. Ring Finger Protein 213 (RNF213), initially identified as the principal susceptibility gene for moyamoya disease (MMD), has emerged as a central regulator of panvascular pathophysiology. This review synthesizes current evidence elucidating RNF213′s multifaceted roles in vascular homeostasis, spanning its functions as an E3 ubiquitin ligase, mechanosensor, and immune modulator. The “second-hit” hypothesis posits that RNF213 mutations establish a genetic predisposition, while secondary insults—such as infection, hypoxia, or hemodynamic stress—precipitate pathological manifestations. Mechanistically, RNF213 orchestrates critical processes including endothelial integrity, angiogenesis, and inflammatory responses through pathways such as HIF-1α/VEGF, NF-κB, and Wnt signaling. Its dysfunction disrupts vascular remodeling, promotes aberrant smooth muscle proliferation, and exacerbates hypoxia-inflammation cycles, contributing to diverse pathologies ranging from intracranial aneurysms and arterial dissections to pulmonary hypertension and coronary artery disease. Emerging insights into RNF213′s interactions with gut microbiota, lipid metabolism, and epigenetic regulators further underscore its systemic influence. Despite advancements, unresolved questions persist regarding the context-dependent duality of RNF213 variants and organ-specific regulatory mechanisms. This review highlights the imperative for integrated approaches combining genetic, molecular, and environmental perspectives to unravel RNF213′s panvascular roles. We also outline unresolved questions regarding context-dependent effects of RNF213 variants and organ-specific regulatory mechanisms, and discuss potential avenues for future research integrating genetic, molecular, and environmental perspectives to advance understanding of RNF213′s panvascular roles.

## 1. Introduction

Vascular diseases remain a leading cause of morbidity and mortality worldwide [1]. Historically, these disorders have been classified based on the specific organs they afflict—be it the brain, heart, or peripheral tissues. However, emerging evidence reveals a unifying thread: a shared pathophysiological foundation that transcends traditional boundaries, giving rise to the concept of pan-vascular diseases [2]. This paradigm shift has given rise to the concept of “panvascular disease” [3,4,5], a holistic framework that underscores the interconnected nature of vascular pathologies across organs, from intracranial arteries to coronary and peripheral vessels. Clinically, researchers have applied the hemodynamic characteristics and experience of coronary atherosclerosis, such as fractional flow reserve (FFR) and wall shear stress (WSS), to renal and peripheral arteries [6,7,8]. Significant progress has been made in assessing stenosis using these parameters. Based on this theory, the parameters are currently being gradually extended to the study of intracranial atherosclerotic stenosis (ICAS). They can predict the occurrence and risk of cerebrovascular diseases. Central to this concept is the recognition that diverse conditions such as MMD [9], intracranial aneurysms, pulmonary hypertension, and coronary artery disease share common pathophysiological roots, including endothelial dysfunction, aberrant remodeling, and inflammatory dysregulation. Unraveling the molecular underpinnings of these shared mechanisms is critical for advancing therapeutic strategies that transcend organ-specific boundaries.

RNF213 encodes a highly conserved, large protein with E3 ubiquitin ligase activity, widely expressed across tissues such as the endothelium, smooth muscle, and immune cells. First spotlighted for its association with MMD—particularly through the founder mutation p.R4810K (rs112735431), prevalent in East Asian populations—RNF213 has since emerged as a pivotal regulator in a broad spectrum of vascular pathologies. This mutation, carried by an estimated 16 million individuals in East Asia [10], is linked to 80–90% of familial MMD cases [11], yet only a fraction—over 50,000—manifest the disease. This incomplete penetrance underscores the complexity of RNF213′s role, pointing to a “second-hit” hypothesis: while RNF213 mutations confer genetic vulnerability (Table 1), additional environmental triggers—such as infections, inflammation, or hemodynamic stress—are required to precipitate clinical disease. Beyond MMD, RNF213 variants are implicated in an array of conditions, from intracranial large artery stenosis and aneurysms to extracranial vasculopathies like coronary and renal artery diseases, prompting the designation of “RNF213-related vascular diseases” as a distinct clinical entity.

The biological effects of RNF213 are multifaceted. As an E3 ubiquitin ligase, RNF213 regulates protein ubiquitination, host defense, angiogenesis, inflammatory responses, and stress adaptation. It also appears to respond to hypoxia and mechanical forces through signaling networks that include HIF-1α/VEGF, NF-κB, Wnt, and caveolin-1/eNOS pathways. Disruption of RNF213 may impair endothelial barrier integrity, alter angiogenic responses, promote aberrant vascular remodeling, and modulate immune activation. These findings support a model in which RNF213 functions as an integrative regulator linking genetic susceptibility to environmental and vascular stress.

This review synthesizes the current literature on RNF213′s expansive role in pan-vascular diseases, exploring its contributions to genetic susceptibility, biomechanical adaptation, and immune regulation. We examine its molecular mechanisms—from hypoxia-driven angiogenesis to infection-related vascular injury—and integrate these findings into theoretical frameworks such as mechanobiological vascular adaptation and hypoxia-inflammation feedback loops. By integrating genetic insights with environmental contexts, this work underscores the necessity of a holistic approach to studying and managing pan-vascular diseases, while elucidating the interplay between genetic predisposition and environmental modifiers to refine our understanding and treatment of these complex systemic disorders.

**Figure 1:**
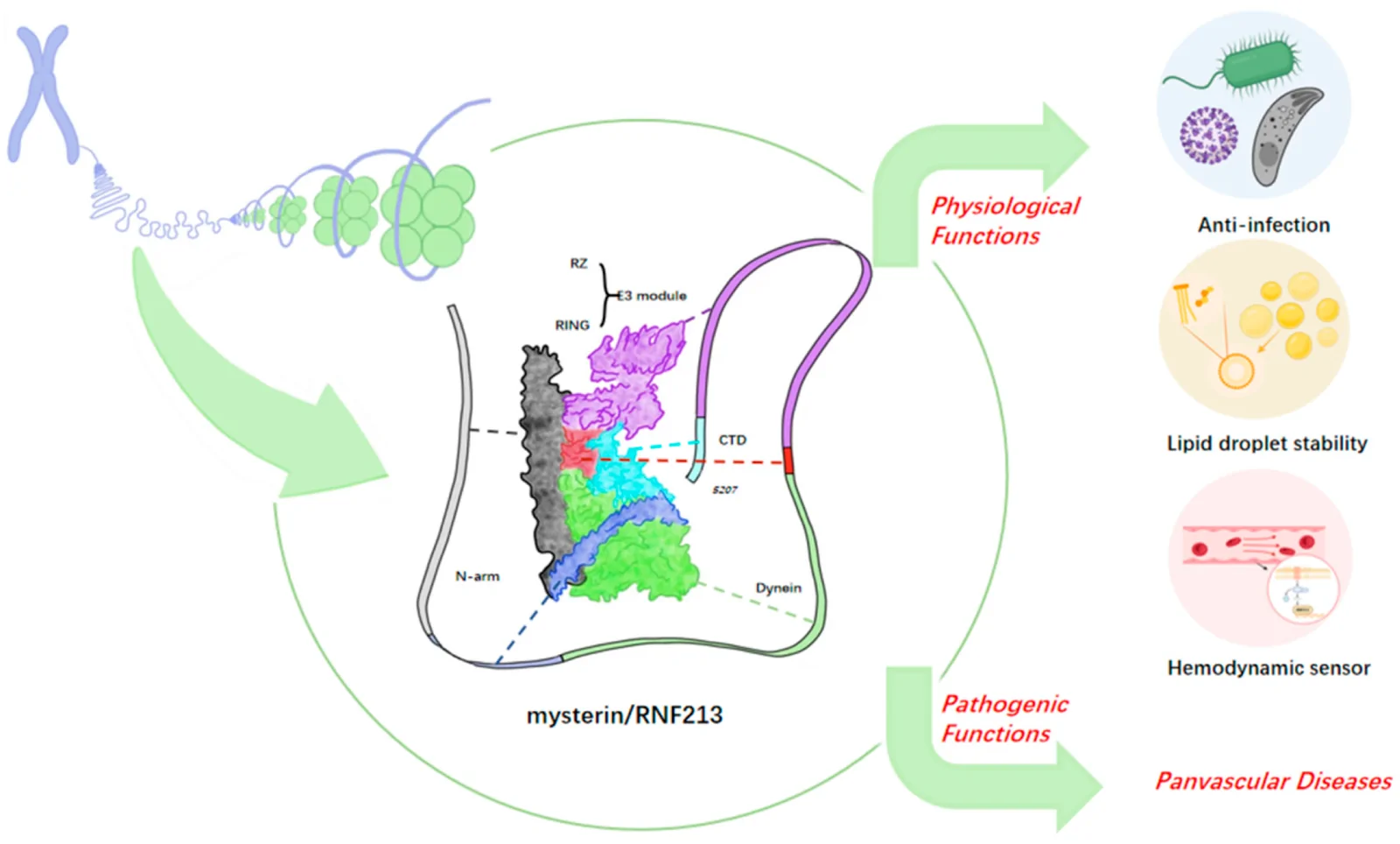
Schematic overview of the RNF213 gene structure, protein domains, physiological functions, and its association with panvascular pathogenesis. RNF213 mRNA undergoes alternative splicing in humans; one variant retains exon 4, while the other exhibits exon 4 skipping [2]. **N-arm**: Located at the N-terminus of the protein, it is involved in transmitting the physical motion generated by the dynein-like ATPase core and maintaining the stability of the molecule. **ATPase core (Dynein):** Comprising six tandem AAA+ modules, it binds and hydrolyzes ATP through its Walker A and Walker B sequences to drive conformational changes in the protein. E3 module: Contains two E3 ubiquitin ligase domains, the RING finger domain and the RZ finger domain. **CTD (C-terminal domain):** Located at the end of the E3 module, it may be involved in regulating the activity of RNF213, particularly in the dynamic changes in the E3 module. Physiological Functions: 1. Antimicrobial defense: Antagonizes bacterial, viral, and parasitic infections. 2. Lipid droplet stabilization and lipotoxicity reduction: Maintains the stability of lipid droplets and reduces lipotoxicity. 3. Blood flow mechanosensing: Acts as a mechanosensor for blood flow, contributing to vascular adaptation. Created in BioRender. Wu, S. (2026) https://BioRender.com/2dhf4o5 (accessed on 7th July 2026).

The spatial structure of RNF213 is dynamically folded due to the conformational driving of its various domains (Figure 1). For instance, the active site of the AAA+ ATPase core can switch between “open” and “closed” states through conformational changes, thereby influencing the overall spatial folding of RNF213. Additionally, upon binding to other substrate proteins, the structure of RNF213 itself undergoes alterations. This multi-domain architecture and dynamic spatial flexibility enable RNF213 to integrate a variety of intracellular signaling changes and adjust its functions in a highly adaptable manner.

### 1.1. RNF213 in Cerebrovascular Diseases

**Figure 2:**
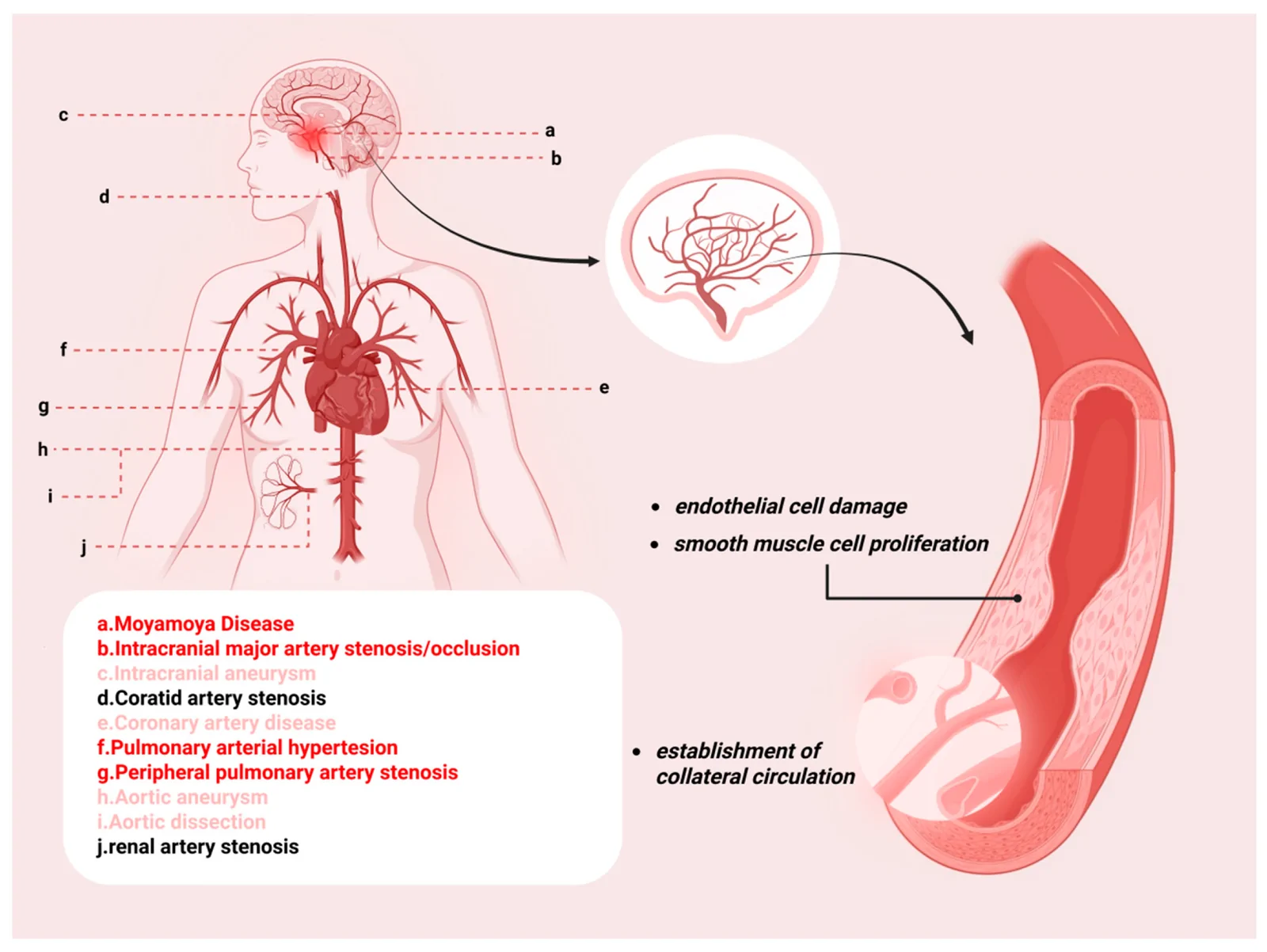
RNF213-associated pan-vasculopathy: Disease spectrum and pathological features. The diseases highlighted in red (a. Moyamoya Disease, b. Intracranial major artery stenosis/occlusion, f. Pulmonary arterial hypertension, g. Peripheral pulmonary artery stenosis) are associated with the RNF213 p.Arg4810Lys mutation; those in light red (c. Intracranial aneurysm, e. Coronary artery disease, h. Aortic aneurysm, i. Aortic dissection) are associated with RNF213 variants; and the diseases in black font are reported to coexist with moyamoya disease (MMD) or RNF213 variants. The common pathological features of MMD and RNF213-related diseases are stenosis caused by smooth muscle proliferation or the establishment of compensatory collateral circulation. This is distinct from the involvement or stenosis of large arteries characterized by atherosclerosis. Created in BioRender. Wu, S. (2026) https://BioRender.com/m39b39v (accessed on 7th July 2026).

The distinct anatomical and physiological characteristics of intracranial arteries—including their abundant web-like collateral circulation, lack of dense connective tissue encapsulation, heightened sensitivity to hemodynamic changes, and smaller diameter with reduced elastic fibers [25] and underdeveloped vasa vasorum (VV) [26]—warrant the establishment of intracranial arterial disease (ICAD) as a distinct clinical entity. This systemic concept encompasses various intracranial vasculopathies that lead to arterial stenosis, including atherosclerosis, moyamoya disease, vasculitis, and arterial dissection [27] (Figure 2).

#### 1.1.1. Moyamoya Disease

Moyamoya disease (MMD) is a complex cerebrovascular disorder pathologically characterized by chronic progressive stenosis or occlusion at the terminal portion of bilateral internal carotid arteries (ICAs) and the proximal segments of anterior cerebral arteries (ACAs) and middle cerebral arteries (MCAs) [28]. Clinically, it is commonly classified into ischemic and hemorrhagic phenotypes [29,30]. The pathological hallmark is concentric fibrocellular intimal hyperplasia [31,32], driven by intimal smooth muscle cell proliferation and extracellular matrix deposition, which produces progressive narrowing of both the vascular lumen and outer arterial diameter [33]. The disease typically presents with idiopathic stenosis/occlusion at the ICA terminus and proximal MCA/ACA segments, accompanied by characteristic compensatory collateral vascular networks [9].

MMD shows marked geographic and ethnic variation, with higher incidence and prevalence in Asian populations. Recent Japanese data report standardized prevalence rates of 14.7–17.6 per 100,000 and annual incidence exceeding 1.8–2.4 per 100,000 [34], whereas reported incidence rates are lower in China [35], Korea [36], North America [37] and Europe [38]. The prevalence among Asian Americans appears closer to that in native Asian populations, supporting a genetic contribution. RNF213 variants also influence clinical phenotype: p.R4810K is more strongly associated with ischemic presentations, whereas p.A4399T has been linked to hemorrhagic disease in Chinese cohorts [14]. Importantly, p.R4810K is mechanistically distinct from complete RNF213 loss: Rnf213 knockout enhances post-ischemic angiogenesis [39], whereas the p.R4810K missense variant exerts anti-angiogenic effects in experimental models [40].

RNF213 also displays a gene-dosage effect [41,42]. Homozygous carriers of c.14576G>A show markedly higher disease penetrance, earlier onset, more severe infarct burden, and poorer outcomes than heterozygous carriers [43,44]. In contrast, heterozygous p.R4810K carriers have low penetrance; population data suggest approximately one MMD case per 150 carriers in Japan [41], and another estimate indicates that only 0.5% of heterozygous carriers develop clinical disease [45]. These observations reinforce the second-hit model, in which immune dysregulation, hypertension, radiation, pharmacological exposure, infection, or other environmental insults interact with RNF213 susceptibility [29]. Notably, smooth muscle cell hyperplasia in MMD may occur independently of RNF213 mutations [46]. CCL5-mediated recruitment of smooth muscle progenitors from endothelial colony-forming cells has been implicated in this process [47], and other susceptibility genes, including ACTA2 [48] and GUCY1A3 [49], also contribute to smooth muscle-related pathology.

Endothelial injury can initiate platelet aggregation and thrombosis. RNF213 variants may influence coagulation through Wnt-related and nitric oxide-dependent pathways. RNF213 deficiency has been associated with increased NFAT1 and filamin A signaling and suppression of Wnt activity [50]. Through β-catenin stabilization, Wnt signaling may facilitate exposure of coagulation factors to subendothelial tissue factor and promote TLR-mediated prothrombotic inflammation [51]. In parallel, eNOS/NO signaling contributes to coagulation homeostasis [52]. Nevertheless, the thrombotic mechanisms linking RNF213 variants to MMD remain incompletely characterized and are likely multifactorial.

Epigenetic mechanisms may further modulate RNF213-associated risk. RNF213 promoter methylation correlates with the p.R4810K variant [53] and shows moderate diagnostic performance for intracranial arterial stenosis [54]. Histone modifications, including H3K4me3 and H3K27ac, regulate RNF213 transcription and may influence variant-associated pathological effects [53,55]. More broadly, moyamoya pathogenesis probably reflects coordinated interactions among multiple genes and pathways, including ACTA2, S1PR1 [56], CECR1, Protein C, Protein S, and GUCY1A3 [41,57].

#### 1.1.2. Intracranial Aneurysms

Intracranial aneurysms (IAs) are pathological dilatations of cerebral arteries whose rupture causes aneurysmal subarachnoid hemorrhage, a neurosurgical emergency. Genetic predisposition [58,59,60] and familial aggregation [61,62] indicate that susceptibility genes contribute to IA/aSAH. High-throughput sequencing has implicated RNF213 variants in IA pathogenesis [17], and mechanistic studies suggest that altered AAA+ ATPase activity may disturb endothelial homeostasis and angiogenic balance, thereby promoting aneurysm formation [15,17,39,63].

Of particular clinical significance, whole-exome sequencing identified a heterozygous RNF213 p.R4810K mutation in a patient who experienced two recurrent intracranial aneurysms following arterial anastomosis [64,65]. This case demonstrates that while bypass procedures and vascular reconstruction can effectively reduce hemodynamic stress through enhanced collateral circulation, RNF213 variants may independently promote aneurysm formation through:

1. Dysregulation of wall shear stress (WSS) distribution patterns [66];
2. Pathological alteration of fundamental hemodynamic profiles [67].

Although RNF213 deficiency may contribute to aneurysm susceptibility, this hypothesis requires validation through functional genomic studies, vascular cell-specific models, and mechanistic analyses of endothelial and mural cell responses.

#### 1.1.3. Intracranial Artery Dissection

Intracranial artery dissection (IAD) represents a rare neurovascular disorder exhibiting lower incidence than cervical artery dissection, with higher prevalence in Asian populations compared to Europeans—a distribution pattern paralleling RNF213 variant frequencies. Despite this epidemiological correlation, the pathogenic mechanisms and genetic basis of IAD remain largely undefined [68]. Known predisposing factors include hypertension, oral contraceptive use, and recent infections [69]. The observed co-occurrence of IAD with moyamoya disease (MMD) suggests these triggers may function similarly to the “second-hit” phenomenon proposed in MMD pathogenesis. IAD is anatomically classified into vertebral artery dissection (VAD) and carotid artery dissection (CAD), with current evidence indicating stronger association between RNF213 variants and CAD. Both familial (f-CeAD) and sporadic (r-CeAD) intracranial dissections have demonstrated RNF213 mutations [70], with one Korean cohort study reporting RNF213 variants in 1/3 of CAD patients, albeit in a limited sample size [22]. While preliminary data suggest RNF213 may influence CAD development [71], its correlation with VAD appears weaker [72]. The unique structural characteristics of intracranial arteries contribute to IAD pathogenesis. Compared to extracranial vessels, intracranial arteries feature: thicker internal elastic lamina, sparse medial elastic fibers and thin adventitia lacking external elastic membrane [73]. The mechanical strength of intracranial arteries primarily derives from the internal elastic lamina. Progressive degradation and weakening of this layer under chronic hemodynamic stress and aging processes may lead to blood intrusion through the vessel wall, potentially developing into intramural hematoma [74,75]. Two distinct dissection subtypes can be identified based on the depth and location of blood penetration [76]. Molecular analyses implicate ICAM-1 adhesion molecules and VEGF signaling in carotid dissections, with notable upregulation of Wnt signaling pathways [77]. RNF213 may contribute to vascular wall disruption through modulation of matrix metalloproteinases (MMPs); tight junction proteins; inflammatory signaling pathways; and extracellular matrix maintenance.

#### 1.1.4. Intracranial Large Artery Stenosis/Occlusion

The RNF213 p.R4810K missense mutation demonstrates significant association with non-MMD intracranial atherosclerotic occlusion (ICASO), with approximately 21.9–24.3% of affected patients carrying this variant [4]. Sequential studies by Miyawaki et al. involving different cohort sizes [16,78], along with other independent investigations [79], consistently indicate that the RNF213 p.R4810K variant specifically associates with anterior circulation ICASO, showing no correlation with posterior circulation ICASO or extracranial carotid atherosclerosis [24].

Similar to moyamoya disease, ICASO exhibits geographical variation in both disease incidence and susceptibility to the p.R4810K variant, as evidenced by pooled odds ratios of 5.59 in Chinese, 28.52 in Korean, and 10.71 in Japanese populations [80]. As of 2018, no other RNF213 variants beyond p.R4810K had been conclusively linked to ICASO [81]. Notably, this particular variant does not appear to affect ubiquitin ligase activity or protein abundance [12], suggesting that disease pathogenesis may instead stem from functional alterations in either the AAA+ domain or N-terminal region of RNF213. Subsequent sequencing analyses have identified novel candidate variants (p.Ser193Gly, p.Val1817Leu, p.Asp3329Tyr, p.Cys118Arg, and p.Leu2356Phe), some of which show exclusive association with ICAS [82]. These findings further substantiate the critical regulatory role of interactions between the N-terminal and AAA+ domain regions in ICAS development, while revealing spatial distinctions from mutation patterns observed in moyamoya disease patients.

### 1.2. Coronary Artery Disease

Coronary artery disease (CAD) is classically linked to lipid metabolism and atherosclerotic coronary stenosis [83], exemplified by variants such as PCSK9 p.E32K, which promotes LDL receptor degradation and hypercholesterolemia [84]. However, RNF213-associated coronary pathology may represent a partially non-atherosclerotic mechanism. Pathological studies of MMD-related intracranial and extracranial vasculopathy, including coronary involvement, often show limited immune cell infiltration and lipid deposition [23]. Two large-scale GWAS analyses have identified associations between RNF213 p.R4810K and coronary phenotypes, including CAD, vasospastic angina, and acute myocardial infarction [23,85]. The underlying mechanisms remain unclear, but the RNF213/Wnt axis may be relevant [86]. Wnt/calcineurin signaling is required for cardiac and coronary development; for example, WNT9b regulates epicardial formation and myocardial growth through β-catenin during coronary vasculogenesis [87]. RNF213 may also modulate eNOS/NO [88], endothelin-1 [89], aldehyde dehydrogenase [90], and caveolin-1-dependent pathways involved in coronary vasomotor regulation. A reported case carrying both RNF213 p.R4810K and PCSK9 p.E32K presented with multiple intracranial arterial stenoses, although lipid deposition and cardiovascular involvement were not fully defined [91]. Overall, RNF213 may define a vascular susceptibility pathway that intersects with, but is not limited to, classical atherosclerotic mechanisms.

### 1.3. Pulmonary Artery Stenosis/Hypertension, Renal Artery Stenosis/Hypertension, and Other Peripheral Arterial Diseases

Associations between MMD and peripheral arterial lesions have been described in pancreatic [92], cervical, mesenteric [93], internal iliac, femoral, and abdominal aortic arteries [94,95]. The best-studied extracranial manifestations are renal artery stenosis/hypertension and pulmonary artery stenosis/hypertension. Renovascular hypertension may be unilateral or bilateral and commonly involves the proximal third of the main renal artery. In MMD-associated renovascular disease, most data are from pediatric populations [96], and combined renal artery stenosis and MMD have been associated with earlier onset and more advanced Suzuki stages [97]. Shared genetic factors, including Notch, ACTA2, and GUCY1A3 [98,99], may partly explain this overlap. A Korean study further implicated RNF213 p.R4810K, reporting an odds ratio of 8.3 for renovascular hypertension among homozygous MMD patients [20]. Although mechanistic and histopathological data remain limited, renal arterial changes appear to resemble those observed in intracranial MMD lesions [100,101,102]. RNF213 has also been associated with pulmonary arterial hypertension (PAH) [103,104], and p.Arg4810Lys is enriched in PAH populations [105], reaching approximately 8% in some idiopathic PAH cohorts [19]. Experimental studies suggest that RNF213 variants contribute to hypoxia-induced pulmonary hypertension in mice [106]. Potential mechanisms include caveolin-1 deficiency and eNOS dysregulation [107,108,109,110], increased CXCL12-CXCR4 signaling that promotes pulmonary arterial smooth muscle and endothelial cell proliferation and migration [111,,112,113,114], and FOSL1-dependent regulation of angiogenesis in LPS-stimulated human pulmonary microvascular endothelial cells [115].

Beyond renal and pulmonary manifestations, RNF213 mutations have also been implicated in pathological alterations of the skin, liver, and other organs [116]. However, the fundamental nature of variants like p.Arg4810Lys—whether they confer gain-of-function or loss-of-function effects—remains unresolved. Notwithstanding this mechanistic uncertainty, RNF213 undeniably plays a pivotal role in systemic vascular pathogenesis and has emerged as a critical biomarker for risk stratification in panvascular medicine.

## 2. The Biological Functions and Mechanisms of RNF213

### 2.1. RNF213 Modulates the Progression of Vascular Diseases in Hypoxic Microenvironments

Hypoxia represents a pathological state of inadequate tissue oxygenation that frequently coexists with inflammatory processes to drive vascular remodeling and angiogenesis [117]. While hypoxic conditions typically induce compensatory neovascularization [118], this hypoxia-angiogenesis coupling mechanism is subject to modulation by RNF213 [40]. Protein tyrosine phosphatase 1B (PTP1B), encoded by the PTPN1 gene and activated by TNF-α [119], participates in vascular maturation and stabilization through its dephosphorylation activity targeting multiple receptors including PDGFR, FGFR, EGFR, HGFR, as well as the angiopoietin receptors Tie1 and Tie2 [120,121]. Crucially, PTP1B serves as a key upstream regulator of RNF213, collaborating with Abelson tyrosine kinases (ABL1/2) via phosphorylation/dephosphorylation dynamics to modulate vascular endothelial physiology under hypoxic conditions. Specifically, hypoxia induces PTP1B-mediated dephosphorylation of RNF213 at Tyr-1275, promoting oligomerization of the RZ domain and consequent enhancement of E3 ubiquitin ligase activity [119]. The activated E3 ubiquitin ligase triggers cell death through two established pathways: first, via CYLD/SPATA2 ubiquitination and degradation that amplifies NF-κB activity, leading to lysosomal degradation and pyroptosis [119,122]; second, through upregulation of α-ketoglutarate-dependent dioxygenase (α-KGDD) activity that increases non-mitochondrial oxygen consumption (NMOC), ultimately accelerating cellular oxygen depletion and cell death [123]. Cellular debris released during this process synergizes with NF-κB activation from hypoxia-induced endoplasmic reticulum stress, collectively promoting NLRP3 inflammasome assembly and subsequent IL-18/IL-1β secretion [124], thereby establishing a self-perpetuating hypoxia-inflammation cycle.

RNF213 serves as a central regulator in these processes, additionally modulating the bFGF/HIF-α/VEGF signaling axis under hypoxic conditions to influence immune infiltration, extracellular matrix remodeling, vascular wall maturation, and pathological intimal hyperplasia in moyamoya disease patients [31]. Experimental evidence demonstrates elevated bFGF (basic fibroblast growth factor) levels in cerebrospinal fluid of both RNF213-knockout mice and MMD patients [125,126]. Through paracrine action on target cells, bFGF stimulates endothelial proliferation and capillary formation [127], while receptor activation induces matrix metalloproteinase (MMP) production in vascular endothelial cells [125]. These MMPs degrade basement membrane components, facilitating recruitment of hematopoietic progenitor cells and endothelial progenitor cells (EPCs)—potentially initiating the extracellular matrix hyperplasia and compensatory angiogenesis characteristic of MMD [128,129].

Furthermore, bFGF potentiates HIF-α activation to enhance endothelial cell motility and upregulate VEGF expression, promoting proliferation of mesoderm- and neuroectoderm-derived cells as well as smooth muscle cells, which may contribute to the characteristic internal carotid artery (ICA) stenosis and occlusion [130]. Notably, HIF family members participate broadly in panvascular pathologies including pulmonary arterial hypertension (PAH), aortic dissection (AD), and atherosclerosis [131]. HIF-1α, the master transcriptional regulator of hypoxic responses, binds hypoxia-responsive elements (HREs) to control expression of multiple genes including VEGF [130,132], Piezo [133], and AngII [134], mirroring clinical observations of aberrant bFGF/HIF-1α/VEGF expression patterns in MMD patients with RNF213 variants or deficiency.

Notably, the biological consequences of reduced VEGF expression differ substantially between cardiomyocytes and endothelial cells. In cardiomyocytes, VEGF downregulation leads to decreased capillary density, resulting in irreversible hypoxic damage that progresses to cardiac fibrosis and heart failure [132], thereby exacerbating systemic hypoxia. Conversely, in endothelial cells, VEGF normally stimulates Ets-1 (E twenty-six homolog 1) to promote endothelial proliferation and angiogenic phenotype transition [135,136], while also facilitating aortic vasa vasorum (AVV) formation through paracrine mechanisms [137]. Suppression of the HIF/VEGF signaling cascade in vascular endothelium compromises oxygen delivery to the vessel wall, precipitating hypoxic injury and inflammatory responses. This cell type-specific dichotomy underscores the complex, context-dependent roles of VEGF in maintaining cardiovascular homeostasis.

Atherosclerotic plaques represent characteristic hypoxic microenvironments [138], circulating monocytes. This chemotactic process is primarily mediated by monocyte chemoattractant protein-1 (MCP-1) secreted by endothelial cells under inflammatory/hypoxic stress, which simultaneously recruits both monocyte populations and EPCs through ligand–receptor interactions while significantly increasing endothelial barrier permeability [139,140]. Notably, the hypoxic/inflammatory milieu induces TNF-α/NF-κB pathway activation in endothelial cells [141], upregulating multiple adhesion molecules including intercellular adhesion molecule-1 (ICAM-1), vascular cell adhesion molecule-1 (VCAM-1), junctional adhesion molecules (JAMs), and platelet endothelial cell adhesion molecule-1 (PECAM-1) [142]. Monocytes utilize surface integrins to specifically recognize these endothelial adhesion molecules (particularly ICAM-1 and VCAM-1), subsequently participating in vascular remodeling and intramural immune infiltration through paracrine signaling [143] and phagocytic activities [144].

Deficiency of RNF213 leads to systemic downregulation of critical endothelial junctional components, including platelet endothelial cell adhesion molecule-1 (PECAM-1), VE-cadherin, β-catenin, plakoglobin, zonula occludens-1 (ZO-1), and tight junction protein claudin-5 (CLDN5), along with reduced expression of adherens junction proteins [145]. Notably, CLDN5 demonstrates not only brain endothelial-specific expression [146], but also significant presence in pulmonary circulatory [147] and renal vascular endothelia [148]. Furthermore, RNF213-deficient brain endothelial cells exhibit diminished MCP-1 production [145,149], a phenomenon potentially mediated through PECAM-1 suppression [149], though the precise mechanism remains unclear. These molecular alterations collectively impair monocyte–endothelial interactions by disrupting membrane protein-dependent recognition, consequently compromising monocyte infiltration and subsequent angiogenic processes. This pathological cascade ultimately exacerbates vascular hypoxia in patients harboring RNF213 mutations, creating a vicious cycle of impaired vascular repair and sustained ischemic injury.

In summary, hypoxia and inflammation form a vicious pathophysiological cycle through vascular dysfunction, with RNF213 serving dual pivotal roles: modulating angiogenic responses while compromising endothelial barrier integrity, thereby amplifying the ischemia-inflammation cascade. Notably, RNF213 likely exhibits organ-specific regulatory effects—its mutations may impair monocyte-mediated vascular repair by suppressing MCP-1 secretion, while concurrently enhancing vascular smooth muscle contraction via the sGC/cGMP/ROCK pathway [150]. These combined mechanisms underlie the regional pathological hallmarks observed in moyamoya disease, atherosclerosis, and other panvascular disorders. It is reasonable to propose that future investigations prioritize elucidating hypoxic niche-specific mechanisms of RNF213, particularly its crosstalk with HIF-1α and therapeutic targeting of endothelial junctional proteins or oxygen homeostasis pathways. Such research could unveil vessel-type-specific regulatory paradigms and inform novel panvascular therapeutic strategies, including disruption of the inflammatory feedback loop and vascular normalization approaches.

### 2.2. RNF213 Modulates Abnormal Vascular Development via Blood Flow Dynamics

Patients with MMD often show compensatory hemodynamic acceleration, whereas severe Suzuki stage III-IV disease may be accompanied by reduced flow velocity. When increased flow exceeds vasodilatory compensation, endothelial cells experience elevated shear stress [151]. This can trigger a shear stress-endothelin-1 positive feedback loop in which mechanical stress upregulates endothelin-1 [152], promoting sustained vasoconstriction and intimal hyperplasia [153] and thereby reinforcing luminal stenosis. The pathological effects of mechanical stress on vasculature involve multifaceted mechanisms [67,154]. Mechanical stress also acts through MMP-9-dependent extracellular matrix remodeling [155] and dysregulated expression of ICAM-1, MCP-1, and NOS [156,157], which modulate NF-κB-dependent inflammatory cascades [158]. Recent studies assessing arterial tortuosity, branching angles, wall shear stress, molecular factors, hemorheology/viscosity, and vascular wall strength have highlighted RNF213 in vascular mechanobiology [159]. RNF213 expression is increased under high wall shear stress (10 Pa) [160], suggesting its potential function as a novel mechanosensitive gene participating in mechanical signal transduction during MMD vascular remodeling.

Several mechanisms may link RNF213 to endothelial adaptation under hemodynamic stress. First, RNF213 helps maintain blood–brain barrier (BBB) integrity by regulating endothelial junctional complexes [145]. RNF213 deficiency or mutation reduces PECAM-1 expression and disrupts its interaction with VE-cadherin. Under physiological conditions, the PECAM-1/VE-cadherin complex sequesters β-catenin at the membrane, limiting nuclear translocation and pro-inflammatory transcriptional programs. This membrane anchoring also indirectly stabilizes CLDN5, a key tight junction protein required for selective BBB permeability [161,162].

Because CLDN5 is essential for BBB selectivity [163], its deficiency may contribute to BBB leakage and cerebral microhemorrhage in MMD [164]. Barrier dysfunction can disturb cerebral microvascular perfusion [165,166], triggering a pathological cascade of aberrant wall shear stress (WSS). This hemodynamic disturbance exacerbates endothelial barrier permeability and inflammatory infiltration through upregulation of adhesion molecules such as ICAM-1 and MCP-1, thereby establishing a self-perpetuating cycle of vascular injury.

**Figure 3:**
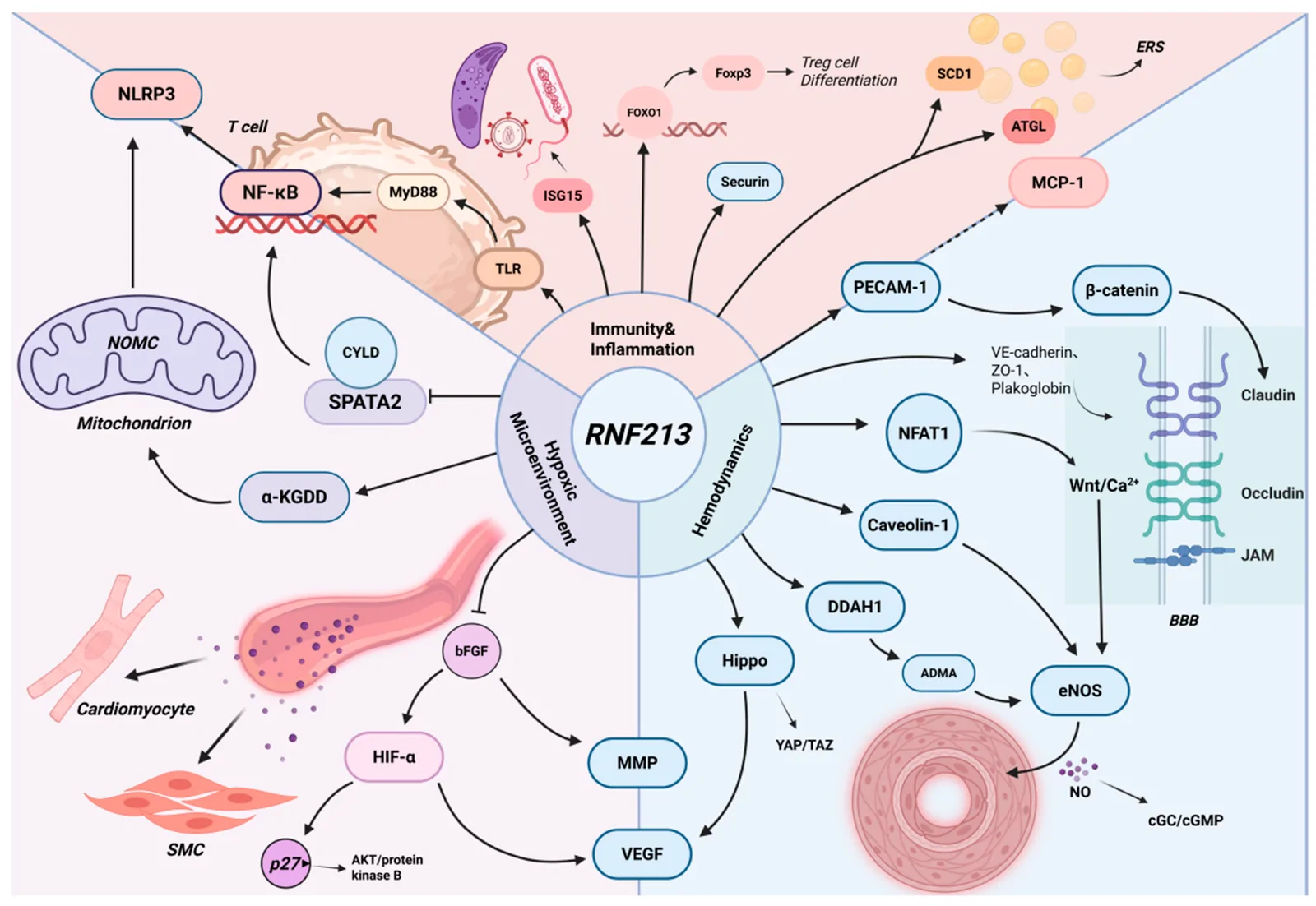
Schematic diagram of the downstream effector molecular network of RNF213. α-KGDD: α-ketoglutarate-dependent dioxygenase; NMOC: non-mitochondrial oxygen consumption; DDAH: dimethylarginine dimethylaminohydrolase; Caveolin-1: caveolin-1; BBB: blood–brain barrier; SPATA2: spermatogenesis-associated protein; CYLD: cylindromatosis protein; bFGF: basic fibroblast growth factor; NFAT: nuclear factor of activated T cells; PECAM-1: platelet and endothelial cell adhesion molecule-1; and ISG15: interferon-stimulated gene 15. Created in BioRender. Wu, S. (2026) https://BioRender.com/6e8d9fi (accessed on 7th July 2026).

(1) RNF213 orchestrates mechanosensitive signaling in endothelial cells through coordinated regulation of membrane protein expression, including PECAM-1, VE-cadherin, and VEGFR2/VEGFR3 [145]. PECAM-1 colocalizes with Piezo1, and together they mediate wall shear stress-induced calcium influx and nitric oxide synthase activation [167]. Piezo channels convert mechanical stimuli into biochemical signals by regulating calcium homeostasis [168] and vascular morphogenesis [169].
(2) Third, RNF213 modulates VEGF expression through Hippo [170] and HIF signaling. VEGF can influence cardiomyocyte function, myocardial blood supply, coronary perfusion efficiency, hemodynamic force, and nitric oxide bioavailability [132].
(3) Endothelial cells regulate the relaxation state of adjacent smooth muscle cells through nitric oxide (NO) [171,172], thereby modulating blood flow velocity and perfusion efficiency. As a key effector of vascular mechanobiology, NO is produced by endothelial cells in response to shear stress [173]. RNF213 may regulate eNOS activity by disrupting inhibitory caveolin-1-eNOS binding [174,175], promoting NFAT1 ubiquitination and degradation to suppress non-canonical Wnt/Ca2+ signaling [50,86], and enhancing DDAH1-mediated removal of ADMA-mediated eNOS inhibition [176,177] (Figure 3). Reduced caveolin-1 protein levels have been reported in MMD and in RNF213 variant carriers [178,179,180], and caveolin-1 dysregulation is also implicated in CAD [181,182] and PAH [110,183], supporting the importance of the RNF213/caveolin-1 axis in vascular pathology and angiogenesis [184,185]. Calcium/calmodulin can displace caveolin-1 from eNOS and thereby promote calcium-dependent eNOS activation [186]. NFAT1 also influences caveolin-1 expression and non-canonical Wnt/Ca2+ signaling, pathways involved in endothelial proliferation and vascular network formation [187,188]. RNF213 deficiency-induced NFAT1 nuclear translocation may reduce vascular regression and contribute to moyamoya vascular abnormalities [86]. NFAT1 overexpression has also been linked to MDM2 activation, p53 degradation, and hepatocellular carcinogenesis [189], indicating broader biological relevance.

DDAH1, which metabolizes ADMA, also participates in antimicrobial defense, neural repair, neurogenesis [190], cardiotoxin metabolism [191], and pulmonary injury responses [192]. By converting ADMA to L-citrulline and dimethylamine, DDAH1 relieves ADMA-mediated eNOS inhibition [176]. eNOS is expressed not only in endothelial cells and cardiomyocytes but also in specialized pacemaker tissues [193]. NO regulates vascular biology through smooth muscle signaling via the NO/sGC/cGMP pathway [194], reaction with superoxide to form peroxynitrite [195,196,197,198], mediation of post-ischemic collateral circulation and vasoactivity [199,200], and maintenance of vascular tone and pressure homeostasis [201,202]. Disruption of these processes can alter vascular tension and hemodynamics [203] and contribute to hypertension, atherosclerosis, stroke, and circulatory failure [204,205,206].

### 2.3. RNF213 Participates in the Regulation of Vascular Inflammation and Immune Homeostasis

Epidemiological studies have demonstrated significant clinical associations between moyamoya disease (MMD) and various autoimmune disorders, including Graves’ disease [207,208], type 1 diabetes [209], thyroid dysfunction or elevated autoantibody levels [209,210,211], systemic lupus erythematosus (SLE) [212], Sjögren’s syndrome, myasthenia gravis, and rheumatoid arthritis [213,214].

Autoimmune conditions such as SLE [215] and antiphospholipid syndrome (APS) [216] can produce occlusive vasculopathy, suggesting overlapping mechanisms of immune-mediated vascular injury and endothelial dysfunction.

Histopathological studies of MMD show absent or minimal inflammatory infiltration in many cases [217,218], with occasional sparse macrophage and T cell infiltration in the superficial thickened intima, potentially related to smooth muscle cell hyperproliferation [219]. Paradoxically, peripheral blood mononuclear cell profiling reveals marked immunodysregulation in MMD patients, characterized by abnormally elevated systemic immune-inflammation index (SII) [220], impaired NK cell maturation accompanied by decreased dendritic cell (DC), effector T cell and stable regulatory T cell (Treg) proportions, alongside significantly increased monocyte populations [221,222]. This immune imbalance may originate from DC-mediated antigen presentation dysfunction, as recent knockout models demonstrate that RNF213 deficiency disrupts DC endosomal systems, manifesting as downregulated expression of early endosome marker Rab5a, late endosome marker Rab7a [223], and lysosomal marker LAMP-1, while substantially inhibiting T cell proliferative capacity both in vitro and in vivo. Given the established role of DCs in cardiovascular diseases, it has been previously proposed that RNF213 plays a pivotal regulatory role in innate immune responses [224], potentially bridging the observed discrepancy between minimal local vascular inflammation and systemic immune dysregulation in MMD pathogenesis.

In the realm of adaptive immune regulation, regulatory T cells, as a subset of CD4+ T cells, maintain immune tolerance through the secretion of TGF-β and IL-10 [225,226]. Foxp3 is a protein molecule that is uniquely expressed in Treg cells [227]. Forkhead box protein O1/3a (FOXO1/3a) plays a crucial role in the early differentiation of the Treg cell lineage, with the nuclear localization of FOXO1/3a promoting Foxp3 expression and enhancing Treg cell stability. RNF213 facilitates the nuclear translocation of FOXO1 via K63-linked ubiquitination, thereby promoting the differentiation of regulatory T (Treg) cells within CD4+ T cells and attenuating the development of autoimmune diseases in a FOXO1-dependent manner [228,229]. This regulatory axis is essential for maintaining Treg cell stability and suppressing autoimmune responses. Notably, Tregs can also modulate the proliferation-apoptosis balance of endothelial cells (ECs) through the TNFR1/Notch signaling axis, thereby influencing the stability of angiogenesis [230]. Clinical studies have confirmed that mutations in RNF213 can lead to impaired Treg differentiation, triggering inflammatory cascades and vascular premature aging [231].

In the context of vascular inflammation, the involvement of immune cells is highly complex. NF-κB is a key regulator of inflammatory signaling, and it has been established that RNF213 can modulate NF-κB activity through multiple downstream pathways. In the vascular walls of patients with moyamoya disease (MMD), abnormal proliferation of smooth muscle cells (SMCs) is frequently observed, accompanied by infiltration of macrophages and T cells [219]. Immunohistochemical analysis has revealed that SMCs migrating into the intima exhibit aberrant expression of IgG and S100A4, suggesting that immune complex deposition may facilitate the transmigration of SMCs through the internal elastic lamina into the intima [232]. This process ultimately leads to intimal thickening and stenosis of intracranial arteries. Notably, M2-type microglia have been shown to exacerbate the production of reactive oxygen species (ROS) and inflammatory responses by downregulating RNF213 expression [233], thereby contributing to the establishment of a chronic inflammatory microenvironment within the vascular wall.

Investigations into the regulatory network of RNF213 on macrophages have revealed that endothelial-specific RNF213 knockdown significantly alters the expression profile of adhesion molecules: upregulation of SELE and VCAM-1, and downregulation of SELP and ICAM-1 [160]. These molecules are dynamically regulated and play critical roles in leukocyte capture, rolling, and transmigration [234,235,236]. Intriguingly, leukocyte (e.g., macrophage) migration is reduced following RNF213 knockdown (RNF213KD), but is markedly enhanced under low-dose LPS stimulation, indicating that RNF213KD sensitizes endothelial cells to inflammatory stimuli [160]. This is consistent with the clinical feature of peripheral blood CD163+ macrophage expansion in MMD patients [222]. CD163+ macrophages mediate various signaling pathways in vascular diseases and contribute to angiogenesis and vascular stability [237]. In atherosclerosis and other vascular diseases, this subpopulation influences plaque stability through pro-angiogenic effects [238].

From a pathophysiological perspective, vascular remodeling is the result of the combined effects of endothelial injury, smooth muscle cell (SMC) proliferation, chronic inflammation, and oxidative stress [57,231]. Chronic inflammation perpetually activates the immune system, with immune cells—particularly T cells and macrophages—and their secreted pro-inflammatory cytokines amplifying the immune response through positive feedback loops, thereby causing endothelial injury. Following endothelial cell injury, SMCs within the vascular wall proliferate and migrate. However, excessive proliferation and collagen deposition can lead to luminal narrowing, ultimately exacerbating vascular stiffness and functional impairment [219]. Cellular rupture and oxidative stress, in turn, promote inflammation and release reactive oxygen species (ROS) [239]. RNF213 plays a pivotal role in this complex regulatory process: Pro-inflammatory cytokines such as TNF-α and IFNγ activate the transcription of RNF213 [240]. RNF213 can subsequently activate NF-κB via multiple pathways [122,240,241], which leads to endoplasmic reticulum stress and the release of the inflammasome NLRP. RNF213 also promotes the expression of matrix metalloproteinases (MMPs) [240], which are key enzymes in the degradation of the extracellular matrix. Importantly, RNF213 mutations can directly cause endothelial cell injury independent of immune cell infiltration [242]. Additionally, RNF213 can modulate the proportion of palmitate in lipid droplets [243], thereby stabilizing lipid droplet stability and reducing lipotoxicity [244], which in turn decreases cell apoptosis and the production of pro-inflammatory substances. Loss of RNF213 function promotes SMC proliferation via the Hippo signaling pathway [170]. Collectively, these findings delineate a molecular framework through which RNF213 mutations lead to a cascade of vascular inflammation and amplification.

### 2.4. RNF213 Contributes to Panvascular Diseases via Its Antimicrobial Activity Against Bacterial, Viral, and Parasitic Infections

Epidemiological studies suggest potential associations between various pathogen infections and cerebrovascular diseases. These include both neurotropic viruses such as herpesviruses [245], varicella-zoster virus [246], adenoviruses [247], enteroviruses [248], and influenza A virus [249], as well as bacterial pathogens including *Haemophilus influenzae* [250,251], *Streptococcus pneumoniae* [252], *Mycobacterium tuberculosis* [253], *Propionibacterium acnes* [254], and *Leptospira* spp. [255]. Notably, some patients with bacterial meningitis may subsequently develop moyamoya-like vasculopathy, a process potentially mediated by pathogen-induced chronic vascular inflammation.

RNF213 has antiviral activity against Rift Valley fever virus, although the precise mechanism remains uncertain [256]. As an innate immune effector, RNF213 participates in antimicrobial defense through several mechanisms. First, it mediates broad antiviral responses through an ISG15-dependent pathway [257]. ISG15 is an interferon-stimulated gene product [258,259,260,261,262] that modifies target proteins through the E1 (UBE1L)-E2 (UBCH8)-E3 (HERC5/TRIM25) cascade [263], thereby restricting viral replication. The N-terminal and C-terminal regions of RNF213 [264] can recognize ISGylated proteins and recruit them to lipid droplets through oligomerization, forming antiviral defense complexes. This ISG15-dependent pathway contributes to control of Listeria infection [265], whereas RNF213 also exerts ISG15-independent antiviral effects against HSV-1258 and KSHV [266], suggesting pathogen-specific adaptor mechanisms.

Second, RNF213 restricts pathogen proliferation through mitochondrial quality control and xenophagy-related mechanisms. In *Toxoplasma gondii* infection models [267,268], IFN-γ stimulates RNF213 E3 ligase activity, leading to ubiquitination of parasitophorous vacuole membrane proteins [269], impaired nutrient acquisition, and autophagosome encapsulation. RNF213 deficiency can impair mitophagy, reduce clearance of infection-induced reactive oxygen species, and promote epithelial cell death [270]. RNF213 may also enhance host defense against *Toxoplasma* by activating the NLRP1 inflammasome [271], providing a mechanistic link between infection and vascular inflammation.

Regarding antibacterial defense, RNF213 mediates ubiquitination of bacterial LPS through the linear ubiquitin chain assembly complex (LUBAC) [272], inhibiting proliferation of Gram-negative bacteria like *Salmonella*. This ubiquitination-dependent immune surveillance mechanism functionally complements the ISG15 pathway, collectively forming the host’s molecular defense against intracellular pathogens. Importantly, chronic inflammation induced by persistent infections may promote vascular remodeling through the NF-κB-MMP axis, while RNF213 mutations causing anti-infection functional defects could be significant contributors to infection-associated vascular abnormalities.

While animal models demonstrate that ISG15-mediated antiviral protection against viral-induced cardiac dysfunction requires RNF213 activity [273], direct experimental evidence for RNF213′s role in infection-related vascular pathologies remains limited. A landmark prospective cohort study has established dynamic interactions between infection burden (IB), RNF213 genetic variants, and vasculopathy progression [274]. Individuals carrying pathogenic RNF213 mutations exhibit elevated IB scores, with the association strength between IB and severe intracranial arterial stenosis (e.g., watershed narrowings) significantly surpassing that of genetic factors alone. This suggests infections may amplify genetic susceptibility through immune-vascular crosstalk.

The “two-hit” pathogenesis model of moyamoya disease [275,276] aligns with these observations: RNF213 mutations provide the genetic substrate, while environmental triggers (particularly recurrent infections) drive vascular pathology. Clinical data reveal that repeated infections exacerbate vascular remodeling, and RNF213-deficient individuals exhibit impaired immune surveillance that perpetuates chronic infections, creating a self-reinforcing “infection–inflammation–vascular injury” cycle. These findings offer a novel framework for understanding phenotypic heterogeneity in MMD and related vasculopathies, where pathogen specificity, infection frequency, and host immune status collectively shape disease manifestations.

### 2.5. RNF213 Is Involved in Lipid and Carbohydrate Metabolism, Modulates Lipid Levels and Blood Glucose, and Influences the Progression of Vascular Diseases

Patients with moyamoya arteriopathy show reduced plasma levels of glycosphingolipids, phospholipids, and sphingolipid metabolites, including sphingosine, dihydrosphingosine, sphingosine-1-phosphate (S1P), and dihydrosphingosine-1-phosphate [277]. These findings suggest that altered lipid homeostasis may contribute to vascular remodeling. Depletion of S1P and related pro-angiogenic lipids may impair endothelial migration and lumen formation [278], reduce progenitor cell recruitment to vascular lesions [279,280], and alter CD36-mediated lipid uptake and endothelial metabolic reprogramming.

Several lipid species, including saturated fatty acids [281], very-long-chain saturated fatty acids [282], and sphingomyelins [283], are linked to cardiovascular risk. RNF213 may regulate lipid metabolism through at least two mechanisms. First, it can bind lipid-droplet surface proteins and inhibit adipose triglyceride lipase activity, thereby reducing lipolysis and stabilizing lipid droplets. Second, RNF213 deficiency increases stearoyl-CoA desaturase-1 activity, promoting conversion of palmitate to monounsaturated fatty acids such as oleate for triglyceride storage [284]. This shift reduces toxic intermediates such as phosphatidic acid and diacylglycerol [243]. Because palmitate is a highly lipotoxic saturated fatty acid [285] that activates the IRE1α-XBP1 arm of the unfolded protein response [286,287], induces endoplasmic reticulum stress and apoptosis in multiple cell types [287,288], and promotes ceramide synthesis [289], reactive oxygen species (ROS) overproduction [290], and snoRNA-mediated epigenetic regulation [291], RNF213-dependent lipid-droplet regulation may protect against lipotoxic vascular injury.

Emerging evidence highlights a functionally significant crosstalk between RNF213-mediated lipid regulation and glucose metabolism. The TNFα/NFκB-p65 axis induces PTP1B expression [292,293], which post-translationally stabilizes RNF213 through dephosphorylation modifications, thereby establishing an autoregulatory circuit that constrains pathological lipid accumulation. This molecular interplay exhibits strong pathophysiological relevance to insulin resistance, where diabetes-associated glycolytic impairment—particularly PFKFB3 activity reduction and consequent fructose-2,6-bisphosphate depletion—exacerbates endothelial dysfunction by impairing cellular proliferation and migration [294,295,296], mechanistically explaining the elevated incidence of ischemic stroke and coronary artery disease in diabetic populations. Clinically, moyamoya arteriopathy (MA) patients demonstrate markedly reduced serum succinate levels (a key tricarboxylic acid cycle intermediate) [278], suggesting that mitochondrial metabolic reprogramming may contribute to lipid homeostasis disruption via compromised fatty acid oxidation. While direct evidence positioning RNF213 as a glucose metabolism regulator remains limited [297], its multimodal functions in alleviating lipotoxicity, suppressing ER stress, and modulating inflammatory cascades strongly implicate its role as a metabolic integrator within the lipid–glucose axis [298], providing novel mechanistic insights into the metabolic heterogeneity underlying MA pathogenesis.

### 2.6. RNF213 in Cell Cycle Regulation and Gut Microbiota Interactions

Current understanding of RNF213′s role in cell cycle regulation remains limited. However, recent studies have provided several key insights:

(1) Downregulation of Cell Division-Related Clusters HL: Zhang et al. reported that knockdown of RNF213 (RNF213KD) results in the downregulation of gene clusters associated with cell division and proliferation, as revealed by clustering analysis [160].
(2) Securin (PTTG1) is a critical regulator of cell cycle progression, particularly in preventing the premature separation of sister chromatids and maintaining cell polarity. RNF213 knockout has been shown to downregulate Securin expression, leading to defects in angiogenesis [299,300].
(3) RNF213 has been implicated in increasing genomic instability during mitosis [301].

These observations collectively highlight the potential role of RNF213 in cell cycle regulation and mitosis. Further investigation into the mechanisms underlying these effects is warranted [41].

Research into the gut microbiota–artery axis has never ceased [302] and many bacterial species have been significantly upregulated or downregulated in patients with moyamoya disease (MMD) [303]. For instance, *R. gnavus* has been reported to be associated with intracranial artery disease (ICAD), such as middle cerebral artery occlusion, coronary artery disease, moyamoya disease, systemic lupus erythematosus [304], and Kawasaki disease [305]. *R. gnavus* produces glucorhamnan and TNF-α [306], both of which can upregulate RNF213. Mutations in RNF213 lead to insufficient pathogen elimination, resulting in chronic inflammation. Conversely, the host can also influence the gut microbiota: angiogenic cytokines (e.g., angiopoietin) may shape the composition of the gut microbiota. As an important regulator of bacterial communities, the broader implications of RNF213 in the gut microbiota field warrant further exploration.

Mechanistically, RNF213 deficiency may reduce securin expression, impair chromosome segregation, and increase genomic instability during mitosis, potentially compromising angiogenesis. At the host–microbe interface, RNF213-dependent ubiquitination and ISG15-related pathways may influence intracellular pathogen clearance and inflammatory tone. These observations support a broader framework in which cell cycle dysregulation, antimicrobial defense, and vascular remodeling are interconnected in RNF213-associated disease.

The gut microbiota–vascular axis may provide a gene–environment interface for MMD and related vasculopathies. Microbial products such as glucorhamnan could activate TLR4/MyD88 and NF-κB signaling, while RNF213 variants may impair ubiquitin-dependent pathogen clearance and perpetuate inflammation. Microbial metabolites may also influence endothelial and cell cycle programs through epigenetic mechanisms. Although these hypotheses remain preliminary, they offer a plausible framework for studying phenotypic heterogeneity in RNF213-associated vasculopathy.

## 3. Concluding Remarks and Limitations

RNF213 has also been implicated in intracranial arterial stenosis among patients with cerebral autosomal dominant arteriopathy with subcortical infarcts and leukoencephalopathy (CADASIL) [307]. Fibromuscular dysplasia [33,308] and Down syndrome [309,310] have also been reported in association with RNF213 and may coexist with MMD. When moyamoya-like vasculopathy occurs in the context of conditions such as sickle cell disease, Down syndrome, neurofibromatosis, radiation exposure, or meningitis, it is commonly classified as moyamoya syndrome [30]. In a cohort of patients with neurofibromatosis type 1, the RNF213 c.14576G>A variant was strongly associated with MMD development [311]. Overall, epidemiological evidence strongly supports a role for RNF213 in MMD and related vasculopathies, but the mechanisms connecting RNF213 variants to arterial stenosis remain incompletely defined. This review highlights mechanobiology [151,159], hypoxia–inflammation crosstalk, antimicrobial defense, metabolism, gut microbiota, cell cycle regulation, embryonic development, and epigenetics as key areas for future study.

This review has several limitations. First, the spectrum of RNF213-associated vascular diseases discussed here is not exhaustive. Moreover, while RNF213 is known to be involved in the oncogenesis and progression of certain cancers (e.g., hepatocellular carcinoma), the similarities and shared molecular pathways between tumorigenesis and panvascular disease progression remain unelucidated; this cross-disease functional overlap may complicate the interpretation of RNF213′s specific role in vascular pathologies. Second, the functional differences between RNF213 knockout, knockdown, and disease-associated missense variants are not fully resolved and should not be assumed to be equivalent. Third, many proposed mechanisms are based on experimental or associative evidence and require validation in vascular bed-specific and cell type-specific models. Future studies should clarify variant-specific effects, identify disease-relevant substrates, and define how genetic susceptibility interacts with environmental triggers to produce organ-specific vascular pathology.

## Figures and Tables

**Table 1:** Summary of RNF213 variants associated with panvascular diseases across diverse populations.

Type ^£^	Variant	Author and Year ^§^	Sample Size Case (M) ^££^/ Control (M)	OR/P	Country/Area	Disease	Domain	Study Type
1	c.14427G>A (p.R4810K)	(Liu et al. 2011) [12]	161 (145)/384 (10) 38 (30)/228 (6) 52 (12)/150 (2)	338.9/1.0 × 10^−100^ 135.6/1.0 × 10^−26^ 14.7/1 × 10^−4^	Japan Korea China	MMD	other	Heritage Research
1	c.14576G>A (p.R4859K)	(Kamada et al. 2011) [13]	63 (46)/429 (6)	190.8/1.2 × 10^−43^	Japan	MMD	other	Heritage Research
1	(p.D4013N)	(Liu et al. 2011) [12]	8 (1)/120 (0)	/	Czech	MMD	RING finger	Heritage Research
1	(p.A4399T)	(Wu et al. 2012) [14]	170 (28)/507 (45)	0.008	China	MMD (bleeding phenotype)	other	Case–Control Study
2	c.12343_12345delAAA (p.K4115del)	(Cecchi et al. 2014) [15]	/	/	America	MMD	other	Heritage Research
2	c.1587_1589delCGC (p.A529del)	(Cecchi et al. 2014) [15]	/	/	America	MMD	other	Heritage Research
1	c.14576G>A (p.R4859K)	(Miyawaki et al. 2012) [16]	41 (9)/25 (0)	12.9/0.01	Japan	ICASO	other	Case–Control Study
1	c.7312C>T (p.R2438C)	(Zhou et al. 2016) [17]	/	/	French-Canada	IA	AAA	Heritage Research
1	c.8476G>A (p.A2826T)	(Zhou et al. 2016) [17]	/	/	French-Canada	IA	AAA	Heritage Research
1	c.14427G>A (p.R4810K)	(Momoi et al. 2024) [18]	140 (9)	/	Japan	CTEPH	other	Case–Control Study
1	c.14427G>A (p.R4810K)	(Hiraide et al. 2020) [19]	139 (11)	/	Japan	PAH	other	Case–Control Study
1	c.14427G>A (p.R4810K)	(Kim and Cho 2021) [20]	32 (30)	8.3(RR)	Korea	RVH	other	Case–Control Study
1	c.14427G>A (p.R4810K)	(Kim et al. 2016) [21]	15 (4)/16 (4)	0.045	Korea	MCAD	other	Case–Control Study
2	c.1214_1216delGAG	(Zhou et al. 2016) [17]	/	/	French-Canada	IA	Treacle	Heritage Research
2	c.11415delC	(Zhou et al. 2016) [17]	/	/	French-Canada	IA	other	Heritage Research
1	c.14427G>A (p.R4810K)	(Kim, Lee and Kwon 2018) [22]	24 (8)/24 (1)	14.247/0.018	Korea	CAD	other	Case–Control Study
1	c.14427G>A (p.R4810K)	(Morimoto et al. 2017) [23]	956/716	2.9/0.005	Japan	Coronary artery disease	other	Case–Control Study
1	c.14427G>A (p.R4810K)	(Shinya et al. 2017) [24]	43 (10)/100 (2)	14.8/2.6 × 10^−5^	Japan	Anterior ICAS	other	Case–Control Study

^§^: First identified. ^£^: Type 1 means point mutation; Type 2 stands for frameshift mutations. ^££^: (M) means mutation. IA, intracranial aneurysm; CAD, cervicocerebral artery dissections; MCAD, middle cerebral artery steno-occlusive disease; RVH, renovascular hypertension; ICASO, intracranial major artery stenosis/occlusion; CTEPH, chronic thromboembolic pulmonary hypertension; and PAH, pulmonary arterial hypertension. c.14576G>A/c.14427G>A: these two mutations actually represented the same variant in RNF213 at the genetic level.

## Data Availability

No new data were created or analyzed in this study. Data sharing is not applicable to this article.
